# A novel capnogram analysis to guide ventilation during cardiopulmonary resuscitation: clinical and experimental observations

**DOI:** 10.1186/s13054-022-04156-0

**Published:** 2022-09-23

**Authors:** Arnaud Lesimple, Caroline Fritz, Alice Hutin, Emmanuel Charbonney, Dominique Savary, Stéphane Delisle, Paul Ouellet, Gilles Bronchti, Fanny Lidouren, Thomas Piraino, François Beloncle, Nathan Prouvez, Alexandre Broc, Alain Mercat, Laurent Brochard, Renaud Tissier, Jean-Christophe Richard, the CAVIAR (Cardiac Arrest, Ventilation International Association for Research) Group

**Affiliations:** 1 CNRS, INSERM 1083, MITOVASC, University of Angers, Angers, France; 2 Anesthesia-Intensive Care Department, European Hospital Georges Pompidou APHP, Paris, France; 3 Vent’Lab, Angers University Hospital, University of Angers, Angers, France; 4 Med2Lab, Air Liquide Medical Systems, Antony, France; 5 SAMU of Paris, Necker Hospital, Paris, France; 6 Ecole Nationale Vétérinaire d’Alfort, IMRB, AfterROSC Network, 94700 Maisons-Alfort, France; 7 Hospital Center of University of Montréal, Montreal, QC H2X 0C1 Canada; 8 Anatomy Department, University of Québec at Trois-Rivières, Trois-Rivières, Canada; 9 Emergency Department, University Hospital of Angers, Angers, France; 10 Inserm, EHESP, University of Rennes, Irset (Institut de Recherche en Santé, environnement et travail) - UMR_S 1085, 49000 Angers, France; 11 Department of Family and Emergency Medicine, FCCM University of Montréal, Montreal, QC Canada; 12 Vitalité Health Network, North West Zone, Edmundston, Canada; 13 Univ Paris Est Créteil, INSERM, IMRB, 94010 Créteil, France; 14 St. Michael’s Hospital, Toronto, ON Canada; 15 Medical ICU, Angers University Hospital, University of Angers, Angers, France; 16 Keenan Research Centre for Biomedical Science, Li Ka Shing Knowledge Institute, St. Michael’s Hospital, Toronto, Canada; 17 Interdepartmental Division of Critical Care Medicine, University of Toronto, Toronto, Canada; 18 Critical Care Department, Angers University Hospital, 4 Rue Larrey, 49933 Angers, France

**Keywords:** Cardiopulmonary resuscitation, Thoracic distension, Intrathoracic airway closure, CO_2_ pattern, Cardiac arrest

## Abstract

**Background:**

Cardiopulmonary resuscitation (CPR) decreases lung volume below the functional residual capacity and can generate intrathoracic airway closure. Conversely, large insufflations can induce thoracic distension and jeopardize circulation. The capnogram (CO_2_ signal) obtained during continuous chest compressions can reflect intrathoracic airway closure, and we hypothesized here that it can also indicate thoracic distension.

**Objectives:**

To test whether a specific capnogram may identify thoracic distension during CPR and to assess the impact of thoracic distension on gas exchange and hemodynamics.

**Methods:**

(1) In out-of-hospital cardiac arrest patients, we identified on capnograms three patterns: intrathoracic airway closure, thoracic distension or regular pattern. An algorithm was designed to identify them automatically. (2) To link CO_2_ patterns with ventilation, we conducted three experiments: (i) reproducing the CO_2_ patterns in human cadavers, (ii) assessing the influence of tidal volume and respiratory mechanics on thoracic distension using a mechanical lung model and (iii) exploring the impact of thoracic distension patterns on different circulation parameters during CPR on a pig model.

**Measurements and main results:**

(1) Clinical data: 202 capnograms were collected. Intrathoracic airway closure was present in 35%, thoracic distension in 22% and regular pattern in 43%. (2) Experiments: (i) Higher insufflated volumes reproduced thoracic distension CO_2_ patterns in 5 cadavers. (ii) In the mechanical lung model, thoracic distension patterns were associated with higher volumes and longer time constants. (iii) In six pigs during CPR with various tidal volumes, a CO_2_ pattern of thoracic distension, but not tidal volume per se, was associated with a significant decrease in blood pressure and cerebral perfusion.

**Conclusions:**

During CPR, capnograms reflecting intrathoracic airway closure, thoracic distension or regular pattern can be identified. In the animal experiment, a thoracic distension pattern on the capnogram is associated with a negative impact of ventilation on blood pressure and cerebral perfusion during CPR, not predicted by tidal volume per se.

**Supplementary Information:**

The online version contains supplementary material available at 10.1186/s13054-022-04156-0.

## Introduction

In the management of cardiac arrest, it is recommended to perform high-quality chest compressions [1]. The optimal ventilation strategy during cardiopulmonary resuscitation (CPR) remains to be determined [2]. CO_2_ monitoring is recommended in clinical practice by International guidelines [1, 3]. However, the application of chest compressions during CPR influences CO_2_ waveform and complicates its interpretation [4, 5]. We previously showed that the actual recommended rate and depth of chest compressions are such that CPR tends to operate below the functional residual capacity (FRC) [6]. During each chest decompression, the recoil of the chest creates a negative intrathoracic pressure with a beneficial circulatory effect. We also showed that the reduction of lung volume due to continuous chest compressions can result in “intrathoracic airway closure” that influences the capnogram waveform [7]. We recently identified in out-of-hospital cardiac arrest patients, another capnogram pattern referred to as “thoracic distension,” in which oscillations are not present at the beginning of expiration but appear after a few chest compressions have been generated, while lung volume decreases. We hypothesized that in case of “thoracic distension,” relatively large insufflations place lung volume above the functional residual capacity, therefore losing the inward/inspiratory recoil of the chest and transiently affecting the circulatory effect of decompression by limiting negative recoil pressure, until returning below FRC. The significance of this “thoracic distension” CO_2_ pattern, as representing a potentially harmful condition for circulation, was investigated in the present study.

The objectives of this study were: (i) to design an algorithm permitting to classify and assess the occurrence of the different CO_2_ patterns observed during CPR in a series of out-of-hospital cardiac arrest patients; (ii) to reproduce the CO_2_ pattern associated with thoracic distension on different experimental models; and (iii) to evaluate the impact of a thoracic distension capnogram pattern on ventilation and circulation in pigs during CPR performed with continuous chest compressions.

## Methods

### CO_2_ patterns detection

#### Capnogram classification: the three patterns

**Fig. 1:**
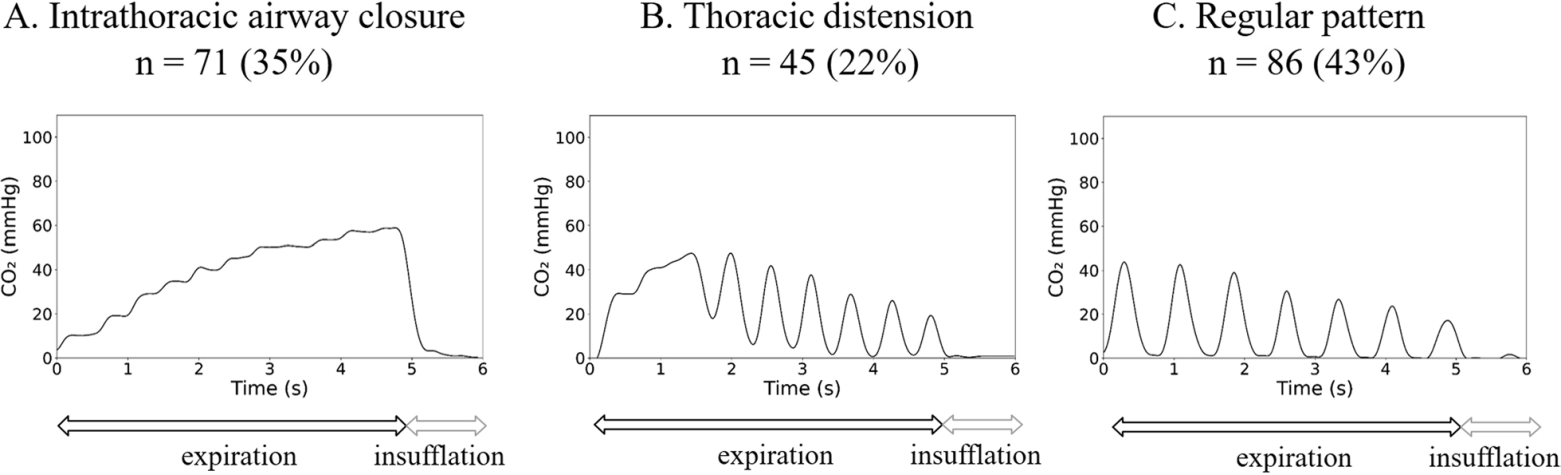
**Capnograms classification from clinical observations**. The figure illustrates the distribution of capnograms according to the classification. Each panel shows a typical CO_2_ pattern obtained from clinical observations after numerical treatment from raw capnogram data (python, Python Software Foundation, Wilmington, Delaware, USA). *X*-axis represents inspiratory and expiratory time. **A** *Intrathoracic airway closure*: oscillations due to chest compressions and decompressions are small or absent. Lung volume reduction far below the FRC and complete or partial intrathoracic airway closure explain this specific capnogram. **B** *Thoracic distension*: oscillations due to chest compressions and decompressions are limited or absent at the beginning of the expiration phase and resume after a few chest compressions. Increase in lung volume due to large *Vt* insufflation before returning to FRC explains this specific capnogram. **C** *Regular pattern*: oscillations due to chest compressions and decompressions are clearly visible during the entire duration of the expiration phase. The regular pattern corresponds to the situation when neither thoracic distension nor intrathoracic airway closure is identified

Capnograms were analyzed as illustrated in Fig. 1 using a simple classification algorithm detailed below. CO_2_ signal obtained with chest compressions during the expiratory phase of the ventilatory cycle was labeled into one of the three patterns defined as follows:

(i) *Intrathoracic airway closure*: oscillations due to chest compressions and decompressions are small or absent. Lung volume reduction far below the FRC and complete or partial intrathoracic airway closure explain this capnogram.
(ii) *Thoracic distension*: oscillations due to chest compressions and decompressions are limited or absent at the beginning of the expiration phase and resume after a few chest compressions. Increase in lung volume above FRC explains this capnogram.
(iii) *Regular pattern:* oscillations due to chest compressions and decompressions are clearly visible during the entire duration of the expiration phase. This pattern corresponds to the situation when neither thoracic distension nor intrathoracic airway closure is identified.

#### Distension ratio, definition and calculation

**Fig. 2:**
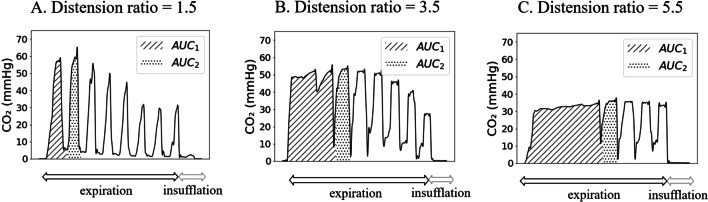
**Quantification of thoracic distension: the distension ratio**. The figure shows examples of capnograms representing different distension ratios (used to quantify thoracic distension) calculated as a continuous variable. Typical capnograms from the animal experiment are displayed for three values of “distension ratio”: 1.5 on panel **A**, 3.5 on panel **B** and 5.5 on panel **C**. *X*-axis corresponds to inspiratory and expiratory time. AUC1 represents the area under the CO_2_ curve between the beginning of the expiratory CO_2_ signal and the first local minimum (The first local minima having an amplitude two times lower than the mean amplitude of all peaks are discarded). AUC2 represents the area under the CO_2_ curve of the first “normal” oscillation corresponding to an efficient compression decompression phase around FRC. The distension ratio corresponds to the ratio AUC_1_/AUC_2_. It is used as a surrogate marker of the level of thoracic distension

To quantify thoracic distension, a distension ratio was defined based on the analysis of the area under the CO_2_ curve (see Fig. 2). In case of thoracic distension, one, two or sometimes more CO_2_ oscillations disappear at the beginning of expiration due to the thorax still transitorily above FRC, preventing the negative recoil pressure during decompression (that only occurs below the FRC). As a result, “thoracic distension” is visible on the capnogram since it prevents several chest compression-induced CO_2_ oscillations. We computed the distension ratio as the ratio between the initial area under the CO_2_ curve without oscillation (AUC1) and the area of the consecutive normal CO_2_ oscillation (AUC2) as illustrated in Fig. 2. A distension ratio of 2 (AUC_1_ is two times superior to AUC_2_) was arbitrarily defined as a cut-off value; considering that the loss of oscillations in case of thoracic distension includes at least two inefficient chest decompressions (distension ratio ≥ 2). Calculations details are available in the Additional file 1: Methods.

#### Capnogram classification: algorithm

1. Maximum and minimum values of CO_2_ peaks corresponding to chest compressions-induced oscillations during the expiratory phase were identified.
2. Airway opening index (AOI) was calculated as defined by Grieco et al. [7] to quantify the magnitude of chest compressions-induced expired CO_2_ oscillations. An AOI lower than 30% was considered as intrathoracic airway closure. The threshold of 30% was defined based on the results of the Grieco et al.’s study showing that below an AOI of 30%, the impact on ventilation and as result on CO_2_ washout was substantial.
3. When AOI was above 30%, the “distension ratio” defined as AUC_1_/AUC_2_ was then calculated (see Fig. 2 and Additional file 1: Methods for details). Thoracic distension was considered when “distension ratio” was greater than 2.
4. Capnogram was considered as regular pattern if AOI was above 30% and distension ratio less than or equal to 2.

### Clinical observations

The main objective of the present clinical series was to confirm the existence of the three CO_2_ patterns at a given time of the CPR process. Capnograms were obtained from patients enrolled in the French RENAU network registry for Out of Hospital Cardiac Arrest (OHCA) (authorization number CNIL 046461). All patients who were receiving manual continuous chest compressions after intubation according to international recommendations [1] with available capnograms (recorded systematically provided there were no technological issues) were consecutively enrolled in the study. Of the patients included in the present study (*n* = 202), capnograms of 89 patients were already reported in a previous study [7]. The CO_2_ pattern was determined based on the classification algorithm described above, using a single representative ventilatory cycle for each patient. Patients were ventilated with a transport ventilator (Monnal T60, Air Liquide Medical Systems Antony, France) using a bilevel pressure mode called CPV, with standardized ventilator settings: respiratory rate (RR) 10 breaths/min; inspiratory time 1 s and expiratory time 5 s (I/*E* = 1/5); inspired oxygen fraction (FiO_2_) 100%; inspiratory pressure 20 cmH_2_O; and positive end-expiratory pressure (PEEP) 5 cmH_2_O. Soon after intubation, CO_2_ signal was recorded and printed at airway opening from LifePak monitor/defibrillator (LIFEPACK 15, Physio-Control, Redmond, WA 98052, USA) with a sidestream sensor placed between the Y-piece and the endotracheal tube. Data were prospectively collected without any interference with care. The study complied with the Declaration of Helsinki and was approved by the ethics committee of the University Hospital of Clermont-Ferrand, France (IRB no. 5891), with waiver of consent.

### Human cadavers with simulation of CO_2_ production

To validate observations obtained from clinical data, the different conditions (i.e., intrathoracic airway closure, thoracic distension and regular pattern) were reproduced with Thiel embalmed human cadavers with simulation of CO_2_ production in the Anatomy Laboratory of the Université Québec à Trois Rivières (UQTR) in Canada with five bodies (authorization number CER-14-201-08-06-17). Those cadavers were validated as a robust model to study ventilation during CPR [8]. The objective of the study was to reproduce on a same human body the three CO2 patterns by changing PEEP and tidal volume. The study was approved by the ethics committee of the University of Quebec at Trois-Rivieres (SCELERA-19-01-PR02). Methods used to ventilate the cadavers and to simulate CO_2_ production have already been described; airway pressure, flow and esophageal pressure were recorded [8, 9] (see Additional file 1: Methods). Manual continuous chest compressions were applied. The airway opening pressure (AOP) was determined in each cadaver as previously reported [10]. Regular pattern and thoracic distension were obtained with a PEEP set above AOP, while intrathoracic airway closure was obtained with a PEEP set below AOP. Using pressure-controlled ventilation, we adapted different inspiratory pressures (20, 30, 40 cmH_2_O) to generate a high range of tidal volumes. Ventilation cycles were classified according to the same algorithm used for the clinical study as intrathoracic airway closure, thoracic distension or regular pattern based only on the CO_2_ signal.

### Mechanical bench with simulation of CO_2_ production

The objective of the bench study was to address the influence of respiratory mechanics and volume on thoracic distension CO_2_ pattern. An original thoracic lung model (POUTAC; non-patented prototype reported in the Grieco et al.’s study [7]) permitting to add a constant production of CO_2_ was used [6] (see Additional file 1: Methods). The model was designed to allow ventilation either above or below FRC (a unique situation specific to CPR) under different combinations of resistance and compliance. Manual chest compressions were applied continuously on the POUTAC using different compliances (*C* = 20–40–60 ml/cmH_2_O) and resistances (*R* = 5–10 cmH_2_O/L/s); capnograms were recorded under a large range of *Vt* (0.3 to 1L). For each combination of *R *× *C* and tidal volume, capnogram was analyzed to detect thoracic distension as described for both the clinical study and cadaver study.

### Animal study

#### Ethical statement

This study was approved by the ethics committee for animal research Cometh-016 (project 2018062813205311). The procedure for the care and killing of study animals was in accordance with the European Community Standards on the Care and Use of Laboratory Animals. A reporting checklist regarding animal preparation and study design is provided in Additional file 2, in compliance with the ARRIVE guidelines.

#### Experimental protocol

We tested 7 female pigs weighing 28 ± 1 kg. A first animal was tested over a large range of tidal volumes (from 6 to 20 ml/kg) to illustrate what can be expected in terms of circulation impact and capnogram patterns.

Six animals were enrolled in the main study. Ventricular fibrillation was induced by a pacing wire inserted in the right ventricular through the femoral vein catheter. Fibrillation was left untreated during 4 min (no-flow period). Then, continuous mechanical chest compression was started at a rate of 100 per minute and a depth of 5 cm with ventilation as recommended (100% oxygen fraction, respiratory rate 10/min, I/*E* 1/5, tidal volume 6 ml/kg). The LUCAS 3™ (Physio-control, Lund, Sweden) chest compression device could exert a mild active decompression effect due to the suction cup. CPR was organized into three periods associated with a specific tidal volume (period T0 to T5 => 5 min at 6 ml/kg-period T5 to T10 => 5 min at 12 ml/kg-period T10 to T15 => 5 min at 6 ml/kg). Blood gases were measured at each tidal volume change. Animals were killed at the end of the protocol (*i.e.,* low-flow period of 15 min) with a lethal dose of pentobarbital (60 mg kg^−1^). Details of animal preparation are available in Additional file 1: Methods.

#### Capnogram analysis and thoracic distension

Thoracic distension was defined based on the “distension ratio” calculated as a continuous variable as illustrated in Fig. 2. This ratio was computed and averaged for each tidal volume period. Correlations between the “distension ratio,” tidal volume, time and hemodynamic parameters were performed.

### Statistical analysis

Statistical analysis was performed with Python Software (Python version 3.9.5, Wilmington—USA). Data are summarized as mean (± SD) for continuous variables and count (%) for categorical variables. In the cadaver experiment, comparisons of tidal volumes between CO_2_ patterns were performed using a repeated measures ANOVA test. Normality of the data was assessed with a Shapiro–Wilk test. Concerning bench experiments, results were averaged over three ventilation cycles for every condition. For the pig experimentation, correlation was assessed using a random effects linear model with each pig’s ID as the random effect. All statistical tests were two-sided, and results with *p* < 0.05 were considered statistically significant.

## Results

### Clinical observations

Capnography was available in 202 patients soon after intubation during chest compressions, and all were included in the study. Patients’ characteristics and outcomes are described in Table 1. Return of spontaneous circulation (ROSC) and rates of survival at hospital admission were 20.5% and 12.9%, respectively.

**Table 1:** Patients characteristics (*n* = 202)

Age (year)	68 (± 15)
Sex male (*n*)	162 (80%)
BMI (kg/m^2^)	25.6 (± 7.2)
Initial rhythm (*n*)	
Non-shockable	153 (73%)
Shockable	57 (27%)
Low-flow time (min)	20 (± 15)
EtCO_2_ at the beginning of ALS (mmHg)	31 (± 18)
Maximal EtCO_2_ during ALS (mmHg)	38 (± 20)
ROSC (*n*)	43 (20.5%)
Survival at hospital admission (*n*)	27 (12.9%)

Data are presented as means (± SD) for continuous variables and count (%) for categorical variables

*BMI* body mass index calculated as weight/height^2^, *EtCO*_*2*_ end tidal CO_2_, *ALS* advanced life support, *ROSC* return of spontaneous circulation

From the 202 capnograms included in the study, 35% showed airway closure, 22% thoracic distension pattern and 43% regular pattern (see Fig. 1). The mean distention ratio was 2.23 ± 2.19 (median 1.55) for all patients, 4.24 ± 2.61 (median 3.43) for thoracic distension patients and 1.16 ± 0.64 (median 1.00) for regular pattern patients.

### Human Thiel cadavers

**Fig. 3:**
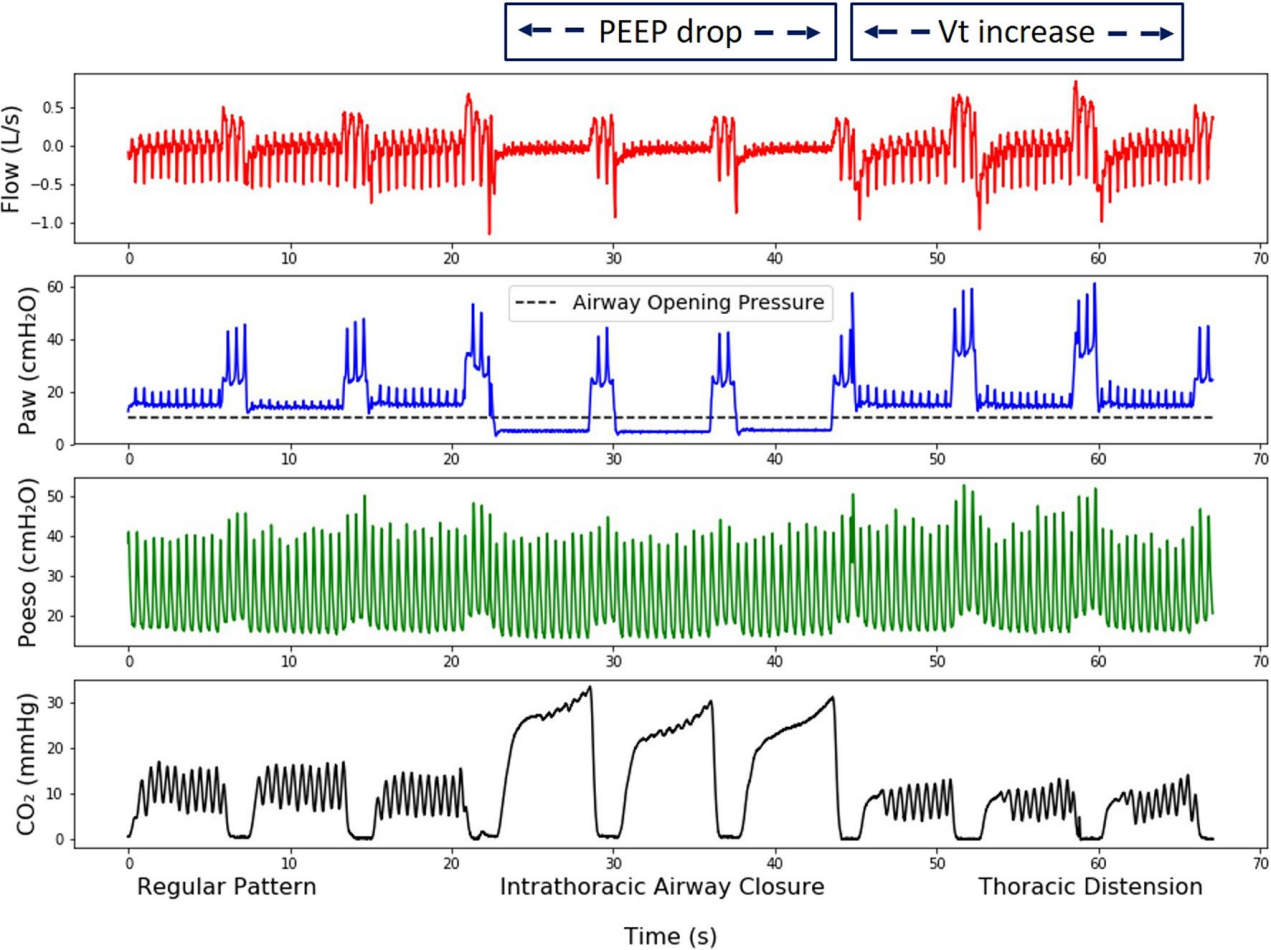
**Reproduction of CO**_**2**_ **patterns on Thiel cadaver model: illustration in one cadaver**. From top to bottom recordings of flow at airway opening (Flow), airway pressure (Paw), esophageal pressure (Peso) and expired CO_2_ (CO_2_). The tilted line on the Paw tracing represents the airway opening pressure (AOP). The recording is divided into three configurations: (1) Regular pattern: positive end-expiratory pressure (PEEP) was set above the AOP to simulate airway patency. (2) Intrathoracic airway closure: PEEP was set below the AOP to simulate airway closure. (3) Thoracic distension: PEEP was set above the AOP to simulate airway patency, and peak airway pressure set on the ventilator was increased to generate higher tidal volumes compared to step 1

The characteristics of the cadavers are given in Additional file 2: Table S1. Figure 3 shows an illustration of the three CO_2_ patterns obtained with the Thiel cadavers. Thoracic distension based on capnogram was associated with higher tidal volumes compared with intrathoracic airway closure (*p* = 0.008) or regular pattern (*p* = 0.005) (after ANOVA). Mean tidal volume was 130 ± 136 ml for intrathoracic airway closure, 453 ± 222 ml for thoracic distension and 141 ± 82 ml for regular pattern. The mean distention ratio was 2.85 ± 1.56 (median 2.65) for all cadavers, 3.72 ± 1.21 (median 3.50) for thoracic distension and 1.23 ± 0.42 (median 1.22) for regular pattern.

### Bench study

Table 2 shows that thoracic distension was favored by high tidal volumes and high time constants (*R* × *C*). The larger the insufflated volume or the longer the time constant, the more likely thoracic distension was present. Thoracic distension was identified on capnograms with a frequency of 0%, 0%, 33%, 33%, 66%, 83%, 83% and 100% for insufflated volumes of, respectively, 300 ml, 400 ml, 500 ml, 600 ml, 700 ml, 800 ml, 900 ml and 1000 ml. Thoracic distension was detected on capnograms with a frequency of 13%, 50%, 38%, 50%, 75% and 75% for *RC* values of, respectively, 0.10 s, 0.2 s, 0.25, 0.40 s, 0.5 and 0.80 s.

**Table 2:**
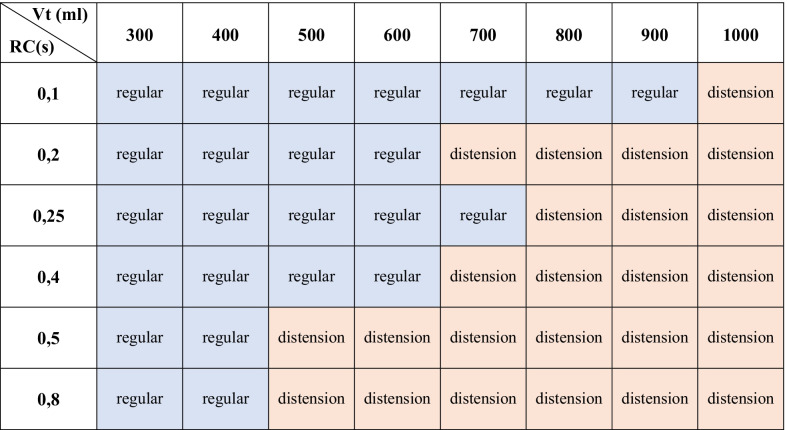
Thoracic distension reproduced on lung model

The thoracic distension pattern was reproduced on the thoracic lung model called POUTAC. This table displays CO_2_ pattern depending on time constant *RC* (multiplication of resistance and compliance) and the set tidal volume using the classification algorithm described in the methods. Each combination of time constant and tidal volume was identified into either regular pattern (called “regular”) or thoracic distension (called “distension”)

*Vt* tidal volume, *RC* time constant corresponding to the multiplication of resistance and compliance

### Pig model

Intrathoracic airway closure was not observed in the animals enrolled in the experiment. Pigs’ characteristics are given in Additional file 2: Table S2.

#### Test animal

**Fig. 4:**
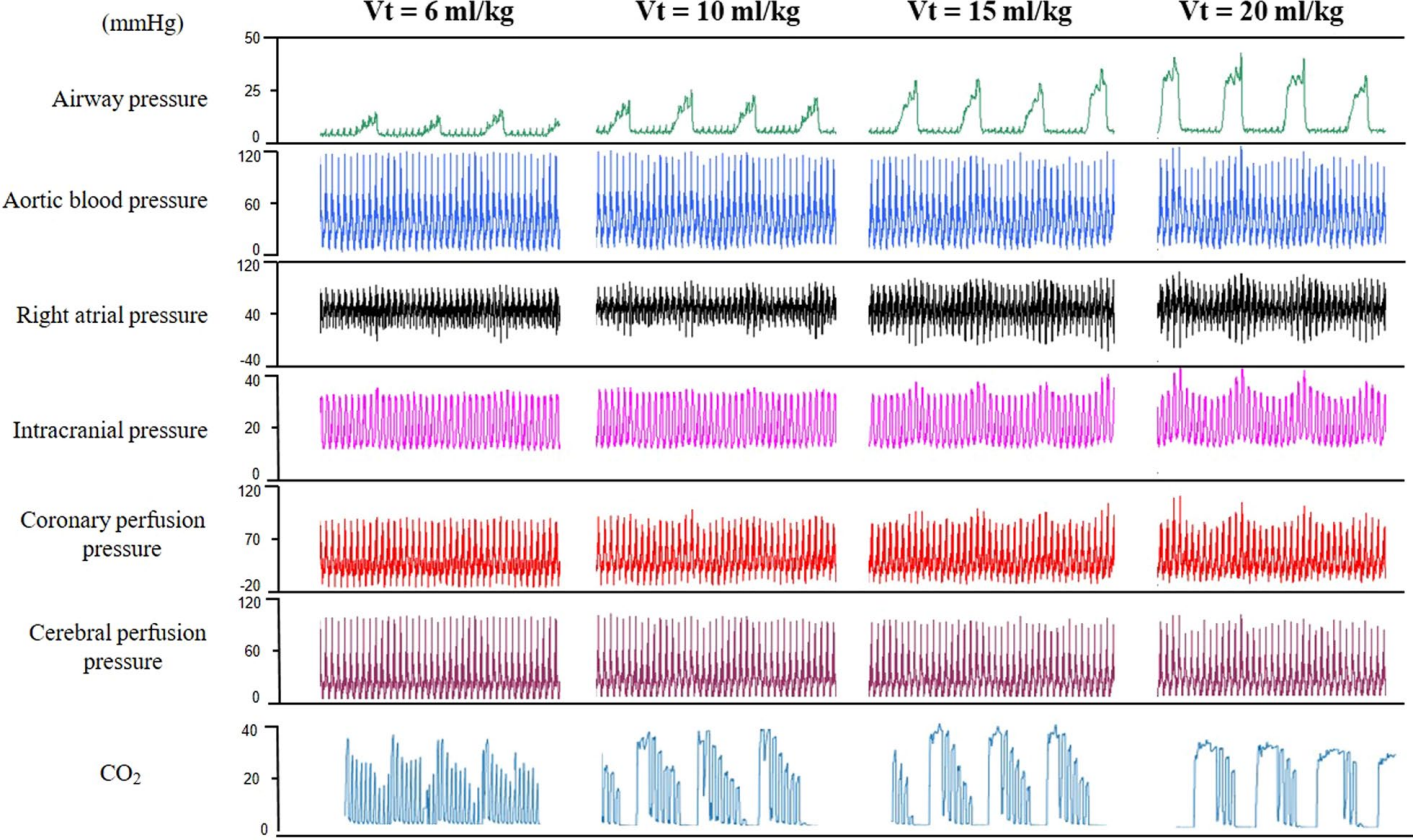
**Impact of a stepwise increase in tidal volume on airway pressure, circulation and capnograms in a pig during cardiopulmonary resuscitation**. From top to bottom, recording tracings of airway pressure, aortic blood pressure, right atrial pressure, intracranial pressure, coronary perfusion pressure (aortic blood pressure minus right atrial pressure), cerebral perfusion pressure (mean arterial pressure minus intracranial pressure) and capnogram during tidal volume (*Vt*) trial. *Vt* was increased as follows: 6–10–15–20 ml/kg. Coronary perfusion pressure waveforms should be interpreted cautiously and read only at end of decompression

Figure 4 illustrates in one animal the increasing variations induced by ventilation of aortic blood pressure, right atrial pressure, intracranial pressure, coronary and cerebral perfusion pressure as *Vt* increased. The capnogram depicted a change of the CO_2_ pattern from regular pattern to thoracic distension as *Vt* increased.

#### Experiment in six animals

**Fig. 5:**
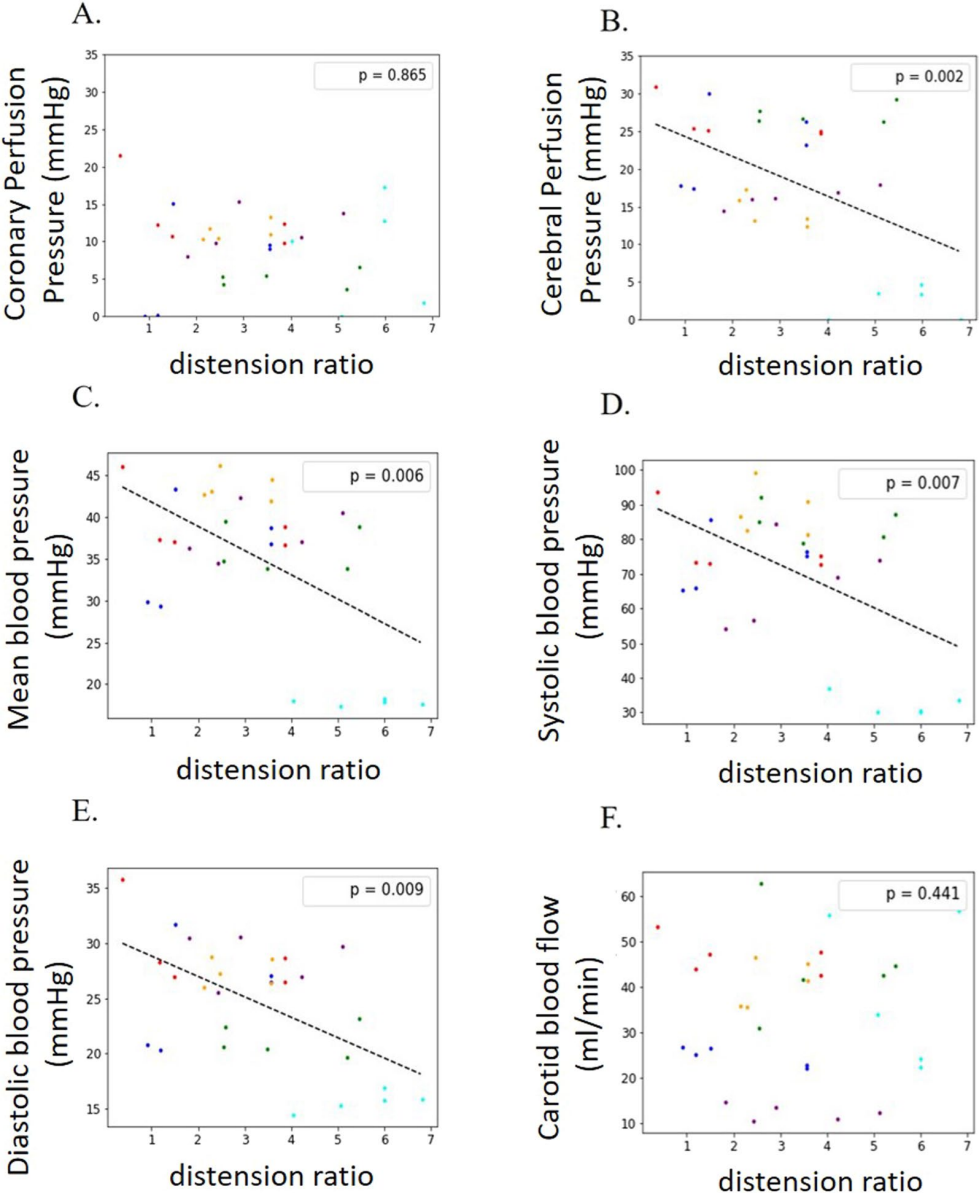
**Relationship between CO**_**2**_ **pattern analyzed by the distension ratio and coronary perfusion, cerebral perfusion, mean, systolic, diastolic blood pressure and carotid blood flow in pigs during cardiopulmonary resuscitation.** **A** Coronary perfusion pressure (measured at end decompression) depending on “distension ratio.” **B** Cerebral perfusion pressure (mean value throughout chest compression/decompression cycles) depending on “distension ratio.” **C** Mean blood pressure depending on “distension ratio.” **D** Systolic blood pressure depending on “distension ratio.” **E** Diastolic blood pressure depending on “distension ratio.” **F** Carotid blood flow depending on “distension ratio.” Correlations were assessed using a mixed linear model. The *p* values are displayed. Each pig is represented by a different color

The “distension ratio,” expressing the level of thoracic distension based on the capnogram (Fig. 5), was significantly and inversely correlated with cerebral perfusion pressure (*p* = 0.002), mean blood pressure (*p* = 0.006), systolic blood pressure (*p* = 0.007) and diastolic blood pressure (*p* = 0.009). There was no significant effect on coronary perfusion pressure and carotid blood flow.

The different hemodynamic parameters recorded were not significantly impacted by tidal volume per se. There was no significant correlation between *Vt* and any recorded circulation parameter: coronary (*p* = 0.283) and cerebral (*p* = 0.998) perfusion pressure, mean (*p* = 0.839), systolic (*p* = 0.962) and diastolic (*p* = 0.882) blood pressure as well as carotid blood flow (*p* = 0.713).

A time effect was present on the different hemodynamic parameters recorded except for coronary perfusion pressure, cerebral perfusion pressure and diastolic blood pressure.

## Discussion

The main results of the present study could be summarized as follows:

1. In the present series of capnograms, intrathoracic airway closure, thoracic distension and regular pattern concerned, respectively, 35%, 22% and 43% of 202 OHCA patients after intubation.
2. The capnogram indicating thoracic distension was associated with higher tidal volumes on Thiel cadavers. Capnogram indicating thoracic distension on a CPR bench model was also more likely to occur with higher insufflated volumes or longer time constants (*R *× *C*).
3. In the animal experiment, the distension ratio calculated from the capnogram to quantify thoracic distension was inversely correlated with cerebral perfusion and arterial blood pressure, while no correlation was found with tidal volume.

### Theoretical optimal thoracic volume for effective chest compressions

The application of continuous chest compressions during CPR complicates CO_2_ waveform interpretation and generates specific CO_2_ patterns [4–7]. Both compression and decompression are needed to generate and sustain effective circulation. The increase in intrathoracic pressure during compression has been shown to generate circulation, thus introducing the concept of thoracic pump theory [11]. Venous return is facilitated by recoil of the chest creating a negative intrathoracic pressure if lung is placed below the functional residual capacity (FRC) when decompression starts. CPR close to the FRC with effective venous return could be identified by the regular CO_2_ pattern with fully oscillating capnogram. Interestingly, non-oscillating capnograms reported by Grieco et al. [7] reflect intrathoracic airway closure that affects ventilation and occurs when thorax is pushed far below the FRC along the course of CPR*.*

### “Thoracic distension” pattern of the capnogram

**Fig. 6:**
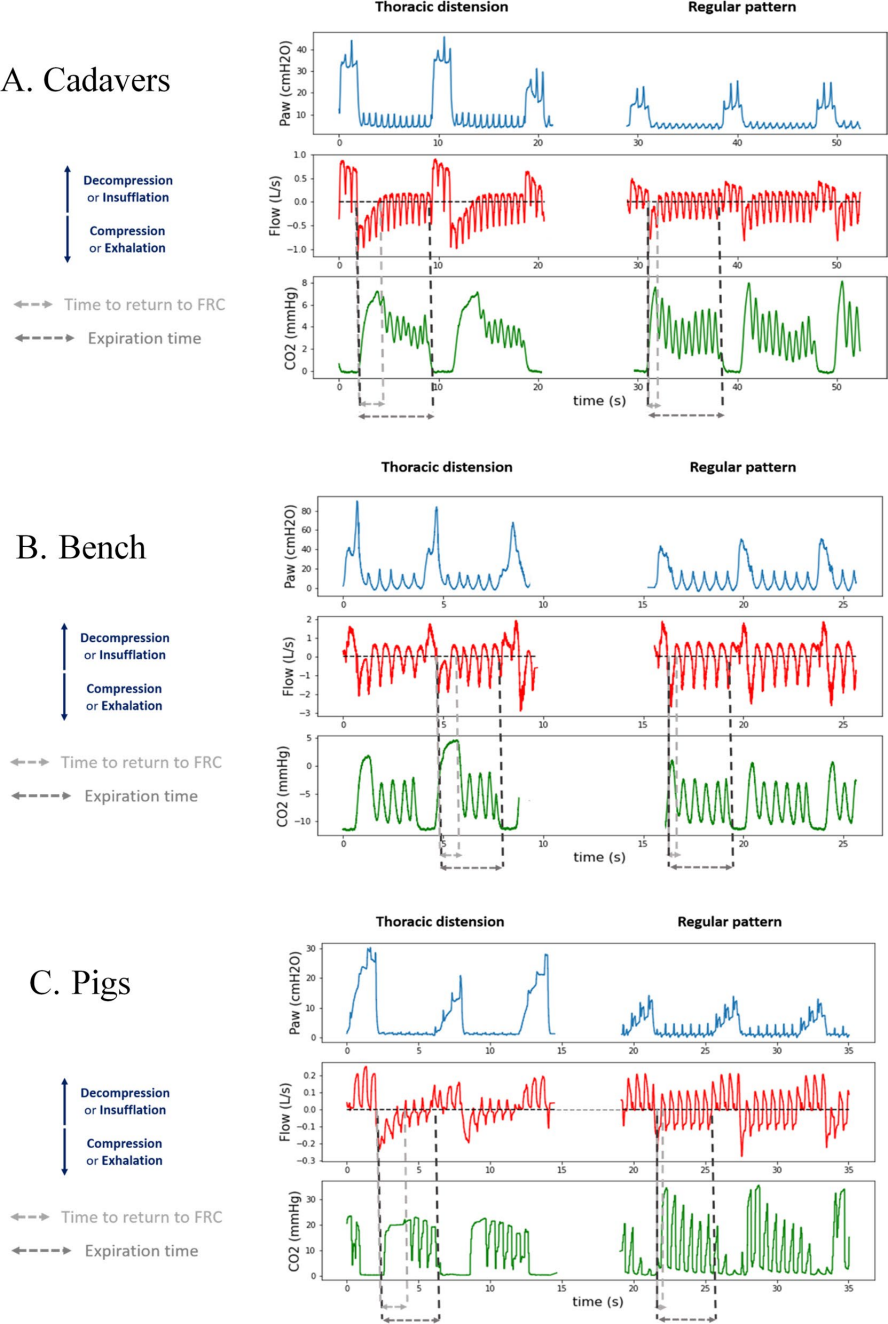
**Illustration of thoracic distension mechanism based on airway pressure, flow and CO**_**2**_ **analysis**. This figure illustrates from top to bottom, airway pressure (Paw), flow at airway opening (Flow) and expired CO_2_ (CO_2_) tracings obtained in cadavers (panel **A**), bench (panel **B**) and animals (panel **C**). The left column illustrates thoracic distension, while the right column represents regular pattern. For each situation, the two gray vertical tilted lines define the time for the lung volume to return to FRC (time with thorax above FRC), while the two black vertical tilted lines define the expiration time (time between two insufflations). Positive flow indicates decompression or insufflation. Negative flow indicates compression or exhalation. Please note the exact time correspondence between flow and CO_2_ oscillations whatever the situation. During expiration, in case of thoracic distension (left column), the flow does not return to zero line during a couple of CC indicating that the thorax is still above FRC even during the decompression phase. CO_2_ oscillations resume only once the flow crosses the zero line, thus indicating the return of lung volume to FRC. On the contrary, the right column obtained with a smaller *Vt* illustrates that the flow induced by CC crosses the zero line immediately after insufflation generating CO_2_ full oscillations. This specific full oscillating CO_2_ pattern indicates that chest compressions operate close to FRC

We hypothesized that the specific capnogram called “thoracic distension” may indicate the risk associated with excessive ventilation inflating the thorax above FRC. It may jeopardize circulation (venous return) by limiting negative intrathoracic pressure during decompression [12, 13]. Expired CO_2_ oscillations which result from the combination of compression and decompression may transiently disappear when the time during which thoracic volume above FRC is prolonged, indicating this risk (see Fig. 6 and Additional file 2).

This is also markedly visible in the pig model (test animal), where we observed that the stepwise increase of *Vt* from 6 to 20 ml/kg magnified coronary and cerebral circulation oscillations related to ventilation and modified capnogram from regular to thoracic distension in parallel (Fig. 4).

### Is the CO_2_ pattern associated with thoracic distension more informative than the ***Vt*** to detect any impact on circulation?

Thoracic distension CO_2_ pattern was reproduced on cadaver, bench and porcine models. This phenomenon was associated on average with higher insufflated volumes compared to intrathoracic airway closure or regular patterns. We found in the pig model that thoracic distension assessed by distension ratio was significantly and negatively correlated with mean arterial blood pressure and cerebral perfusion pressure, suggesting its potential negative impact on circulation during resuscitation.

Unlike the capnogram, *Vt* absolute values were not significantly associated with a negative effect on blood pressure, coronary perfusion and cerebral perfusion. Those results may suggest that the capnogram may be more relevant than *Vt* per se to predict a circulatory impact induced by ventilation.

The bench study provides a possible explanation for the previous observed result. Indeed, prolonged time constant that characterizes the time required to return to FRC may favor thoracic distension even with low *Vt*, as we observed in some animals.

### Occurrence of thoracic distension, intrathoracic airway closure and regular capnogram

In our series of 202 OHCA patients, thoracic distension and intrathoracic airway closure concerned 22% and 35% of patients, respectively. Interestingly, very similar capnograms have been reported during CPR, without specifically identifying the phenomenon of thoracic distension [4, 5].

An important methodological point is that capnograms from the present study were captured soon after intubation with a respiratory rate of 10/min and a protective pressure mode of ventilation limiting *Vt*. One cannot exclude that thoracic distension may be much more frequently observed with manual bag ventilation during which *Vt* and respiratory rate are poorly controlled, thus favoring the risk of hyperventilation. In addition, a moderate level of PEEP was used in our series, which could have minimized the occurrence of intrathoracic airway closure, favored by low airway pressures. Although our brief periods of recordings with one to ten cycles displayed similar patterns for all breaths, it is likely that CO_2_ patterns evolve along the course of CPR, and that the classification could change depending on the time of intervention, thus precluding any interpretation of its significance in terms of outcome.

Of note, intrathoracic airway closure was not observed during the animal experiment. It is possible that the pig thorax anatomy may limit the reduction of lung volumes we observe in humans during resuscitation and thus occurrence of intrathoracic airway closure. Besides, pig bronchial tree presents lateral connections that may also limit occurrence of distal airway closure [14]. In addition, the mechanical chest compression device used in the swine study was operated with a mild active decompression due to the suction cup, which may limit the reduction of lung volume below the FRC potentially responsible for intrathoracic airway closure.

### Clinical perspectives

Excessive ventilation during cardiac arrest has already been shown to be associated with poor outcomes [15, 16]. Nevertheless, it is definitively challenging to control and monitor *Vt* delivered during manual bag ventilation [17].

Based on these observations, a capnogram-based ventilation strategy may permit to optimize ventilation during CPR, using real-time identification of capnograms (intrathoracic airway closure, thoracic distension or regular pattern). As previously shown, PEEP increase may be considered in case of intrathoracic airway closure to open the airways, while *Vt* reduction could be proposed in case of thoracic distension. Further evidence is needed before developing such ventilatory approach on a ventilator, but these findings may be of potential additional value for bag valve mask ventilation during which hyperventilation is likely to occur.

### Study limitations

First, the capnogram analysis proposed in the present study is based on continuous chest compression, and whether it is generalizable to an interrupted chest compression strategy ideally needs further assessment. But thoracic distension may also be present during interrupted chest compressions.

Second, capnogram from one ventilatory cycle recorded soon after intubation (according to the local routine procedure) was analyzed for each patient. This relatively limits the possibility to generalize CO_2_ pattern distribution to different CPR strategies (chest compression frequency, depth or other) and renders hazardous outcomes’ interpretation.

Third, the specific setup in cadavers experiment to administer CO_2_ via a catheter placed in the endotracheal tube resulted in significant additional resistance that favored early occurrence of thoracic distension as suggested by the observations obtained on the bench.

In the animal study, since each animal was its own control, several time-related factors might have also impacted circulation. Further studies comparing animals with different ventilation strategies are needed to confirm our observations.

## Conclusion

During CPR, intrathoracic airway closure, thoracic distension or regular pattern can be reliably identified by the capnogram analysis. We describe a novel CO_2_ pattern indicating relative thoracic distension, which may be associated with a negative impact on blood pressure and cerebral perfusion, irrespective of tidal volume per se. This original capnogram classification has the potential to help optimizing ventilation during CPR.

## Supplementary Information

**Additional file 1: Methods.** Distension ratio: calculation details. Human cadavers with simulation of CO_2_ production. Mechanical bench with simulation of CO_2_ production. Pig study: animal preparation.

**Additional file 2: Results.** Clinical study from Grieco et al. Illustration of thoracic distension mechanism based on airway pressure, flow and CO_2_ analysis. Table S1: Cadavers’ characteristics. Table S2: Baseline pigs’ characteristics. Compliance to the ARRIVE Guidelines of the pigs’ experiment.

## Data Availability

The datasets used and/or analyzed during the current study are available from the corresponding author on reasonable request.

## Abbreviations

CPR: Cardiopulmonary Resuscitation
FRC: Functional Residual Capacity
RR: Respiratory Rate
FiO_2_: Inspired Fraction of Oxygen
PEEP: Positive End-Expiratory Pressure
ROSC: Return Of Spontaneous Circulation
*R*: Resistance
*C*: Compliance
