# Supplementary material for: A novel capnogram analysis to guide ventilation during cardiopulmonary resuscitation: clinical and experimental observations
Source: Crit Care. 2022 Sep 23;26:287. doi: 10.1186/s13054-022-04156-0 (PMC9508761; doi:10.1186/s13054-022-04156-0)
Supplement: Supplementary file 1 — Additional file 1: Methods. Distension ratio: calculation details. Human cadavers with simulation of CO2 production. Mechanical bench with simulation of CO2 production. Pig study: animal preparation. [file 13054_2022_4156_MOESM1_ESM.docx]

**Additional file 1: Methods**

***Distension ratio: calculation details.***

Distension ratio was defined based on the analysis of the area under the CO2 curve in order to quantify thoracic distension. Thoracic distension is characterized by the absence of several CO2 oscillations at the beginning of expiration despite the delivery of chest compressions. This reflects the displacement of the thorax above the Functional Residual Capacity (FRC) due to the insufflation, preventing the negative recoil pressure during decompression (that only occurs below the FRC).

The distension ratio is the ratio between two areas under the capnogram curve (see figure 1):

**1.** The area 1 under the CO2 curve (AUC1) from the beginning of expiration to the first local minimum.

**2.** The area 2 under the CO2 curve (AUC2) of the first “normal” oscillation corresponding to an efficient compression decompression phase around FRC.

Of note, the point of transition from the first oscillation (AUC1) to the first "normal" oscillation (AUC2) corresponds to the first local minimum with one condition:

- The first CO2 peaks (including local minima) with an amplitude much lower than the mean amplitude of all the peaks are discarded from the analysis.
- Amplitude is calculated as $\frac{(CO2\max- CO2 min)}{CO2 max}$ where CO2max and CO2min are respectively the local maximum and minimum CO2 values of each oscillation.

To better illustrate this condition, a capnogram from one ventilatory cycle was displayed in the figure below. The local maxima and minima of chest compressions-induced CO2 peaks are displayed in red points and blue points respectively. In this case, the CO2 peak associated with the first local minimum has a lower amplitude (orange arrow) compared to the mean amplitude of all the peaks. Consequently, this oscillation is discarded from the analysis. The point of transition from the first oscillation (AUC1) to the first "normal" oscillation (AUC2) corresponds to the second local minimum.


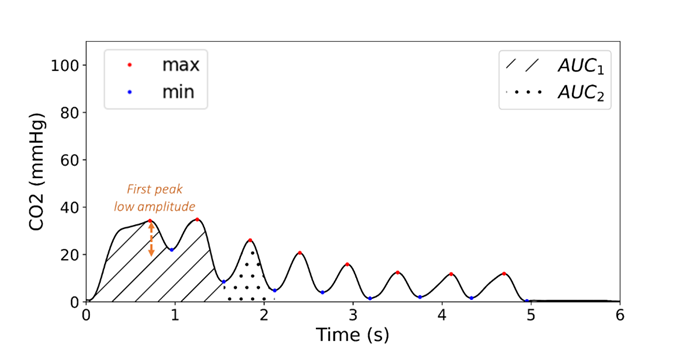


***Human cadavers with simulation of CO_2_ production***

Constant CO_2_ flow was inserted via the intubation probe to simulate CO_2_ production. Data were recorded with Biopac acquisition system. A pneumotachograph (Fleisch n◦2, Lausanne, Switzerland), a pressure transducer (SD160 series: Biopac systems, Goleta, CA, USA) and an infrared-based CO_2_ sensor (CO_2_-100C: Biopac systems, Goleta, CA, USA) were used to measure flow, airway pressure and CO_2_. Signals were converted with an analog digital converter (MP150; Biopac systems, Goleta, CA, USA) at a sample rate of 200Hz, and stored in a laptop using a dedicated software (Acknowledge, Biopac Systems, Goleta, CA, USA). The cadavers were ventilated with an emergency ventilator (Monnal T60, Air Liquide Medical Systems Antony, France) using pressure mode with standardized settings (RR=10 breaths/min, I/E=1/5, PEEP=5 cmH_2_O, FiO_2_ 100%). Airway Opening Pressure (AOP) was assessed using a pressure time curve recorded during a low flow insufflation (10). The AOP was defined as the pressure level where the pressure time curve abruptly changes from a very steep to a smoother slope.

***Mechanical bench with simulation of CO_2_ production***

A thoracic lung model called POUTAC was used to reproduce the mechanical properties of the respiratory system during CPR as described previously (6). The model is designed to allow ventilation either above (as allowed by all lung models) or below FRC (a unique situation specific to CPR). A bellow on which chest compressions can be applied mimics the lung with an adjustable equilibrium volume representing the Functional Residual Capacity (FRC). A wide range of respiratory mechanics (resistance and compliance) can be tailored. By providing CO_2_ to the base of the bellow, our model simulates the production of CO_2_, which allows capnograms to be recorded. An infrared-based CO_2_ sensor (CO_2_-100C: Biopac systems, Goleta, CA, USA) was used to measure CO_2_. During our experiment, ventilation was delivered through an emergency ventilator (Monnal T60, Air Liquide Medical Systems) in Assis Control Ventilation (ACV) mode with a frequency of 10/min and a positive end expiratory pressure (PEEP) of 5 cmH_2_O.

***Pig study: animal preparation****.*

We tested 7 female pigs weighing 28±1 kg, including 1 animal for the initial test and 6 animals for the main study. They were anesthetized with a mixture of tiletamine (10 mg.kg^-1^ i.v.), zolazepam (10 mg.kg^-1^, i.v.), propofol (10 mg.kg^-1^.h^-1^ i.v.) and methadone (0.3 mg/kg^-1^ i.m.). Animals were intubated and mechanically ventilated (Monnal T60, Air Liquide, Antony, France) in ACV mode (30% Oxygen, tidal volume 9 ml/kg, RR=20). Body temperature was controlled for a core temperature at 38°C. Animals were monitored by a five-lead electrocardiogram. Oxygen saturation (SpO_2_) and CO_2_ using the Monnal system (Irma CO_2_ probe Monnal, Masimo Corporation CA, USA) were recorded. Catheters were inserted into femoral artery and vein for the evaluation of aortic and right atrial pressure, respectively. The central catheters were inserted into the femoral artery and vein and then mounted to the aortic and right atrial levels, respectively. Catheters are then positioned at those levels but not by chest opening or laparotomy. An intracranial pressure probe was inserted after trepanation (Millar®, Houston, USA). A flow probe was implanted around the carotid artery for the continuous evaluation of the carotid blood flow (PS-Series Probes, Transonic, NY, USA). After a period of stabilization, animals were paralyzed by rocuronium (1 mg.kg^-1^). The mechanical compression device, a LUCAS 3^TM (^Physio-control, Lund, Sweden) was placed in a controlled and secured position and was operated with default settings. Hemodynamic parameters were continuously recorded throughout the experimental protocol (HEM version 4.2, Notocord, Croissy-sur-Seine, France). Parameters continuously recorded were: airway pressure (mmHg), aortic blood pressure (mmHg), right atrial pressure (mmHg), intracranial pressure (mmHg), carotid blood flow (ml/min) and CO_2_ (mmHg). Coronary perfusion pressure was calculated as aortic blood pressure minus right atrial pressure at end-decompression. Cerebral perfusion pressure was calculated as the mean value of arterial pressure minus intracranial pressure throughout chest compression and decompression cycles.
