# Supplementary material for: A novel capnogram analysis to guide ventilation during cardiopulmonary resuscitation: clinical and experimental observations
Source: Crit Care. 2022 Sep 23;26:287. doi: 10.1186/s13054-022-04156-0 (PMC9508761; doi:10.1186/s13054-022-04156-0)
Supplement: Supplementary file 2 — Additional file 2: Results. Clinical study from Grieco et al. Illustration of thoracic distension mechanism based on airway pressure, flow and CO2 analysis. Table S1: Cadavers’ characteristics. Table S2: Baseline pigs’ characteristics. Compliance to the ARRIVE Guidelines of the pigs’ experiment. [file 13054_2022_4156_MOESM2_ESM.docx]

**Additional file 2: Results**

***Clinical study from Grieco et al.***

Interestingly, we applied the present capnogram classification to the previous series (7). The results are very consistent since they show airway closure 36%, thoracic distention 19%, regular pattern 45% versus 35%, 22%, and 43% respectively in the present entire series. The median value of the AOI in the previous series was 50 % versus 52 % in the present series.

***Illustration of thoracic distension mechanism based on airway pressure, flow and CO2 analysis***

We hypothesized that thoracic distension results from insufflations that place lung volume above functional residual capacity, even at the beginning of expiration. It transiently affects chest decompression by limiting negative recoil pressure, until returning below FRC.

The examination in the animal, bench and cadaver models of concomitant changes of airway flow, airway pressure and CO2 during chest compressions may help to better understand this point regarding the position of the thorax. As illustrated on figure 5, there is an exact correspondence between the flow signal and the CO2.

During expiration, in case of thoracic distension (left column), the flow does not return to zero line during a couple of CC indicating that the thorax is still above FRC (exhalation time) even during the decompression phase. CO2 oscillations resume only once the flow crosses the zero line, thus indicating the return of lung volume to FRC.

On the contrary, the right column obtained with a smaller Vt illustrates that the flow induced by CC crosses the zero line immediately after insufflation generating CO2 full oscillations. This specific full oscillating CO2 pattern indicates that chest compressions operate close to FRC.

**Table S1:** *Cadavers’ characteristics*

| **Age (years)** | **68.6 (± 4.7)** |
| --- | --- |
| **Sex male** | **4 (80%)** |
| **Height (cm)** | **169 (± 9)** |
| **Weight (kg)** | **62.7 (± 9.5)** |

Data are presented as means (**±** SD) for continuous variables and count (%) for categorical variables.

**Table S2:** *Baseline pigs’ characteristics*

| **Weight (kg)** | 28 (± 1) |
| --- | --- |
| **Heart Rate (bpm)** | 87 (± 32) |
| **Systolic blood pressure (mmHg)** | 86 (± 12) |
| **Diastolic blood pressure (mmHg)** | 56 (± 10) |
| **Mean arterial pressure (mmHg)** | 70 (± 11) |
| **Right atrial pressure (mmHg)** | 13 (± 1) |
| **Coronary arterial pressure (mmHg)** | 53 (± 13) |
| **Systolic Intracranial pressure (mmHg)** | 9 (± 6) |
| **Diastolic Intracranial pressure (mmHg)** | 7 (± 6) |
| **Mean Intracranial pressure (mmHg)** | 8 (± 6) |

Data are presented as means (± SD)

**Compliance to the ARRIVE Guidelines of the pigs’ experiment**

| **TITLE** | **A novel capnogram analysis to guide ventilation during continuous chest compressions resuscitation. From clinical to experimental observations** |
| --- | --- |
| **METHODS** |  |
| - *Ethical statement* | This study was approved by the ethics committee for animal research Cometh - 016 (project 2018062813205311). The procedure for the care and sacrifices of study animals was in accordance with the European Community Standards on the Care and Use of Laboratory Animals. |
| - *Study design* | In a first animal, we tested the effect of a large range of tidal volumes during cardiopulmonary resuscitation (from 6 ml/kg to 20 ml/kg) to illustrate what can be expected in terms of circulation impact and capnogram patterns.  The six other animals were enrolled in the main study. Ventricular fibrillation was induced by a pacing wire inserted in the right ventricular through the femoral vein catheter (no specific technical issues were observed during cardiac arrest induction). Fibrillation was left untreated during 4 min (no-flow period). Then continuous mechanical chest compression was started at a rate of 100 per minute and a depth of 5 cm with ventilation as recommended (100 % oxygen fraction, respiratory rate 10/min, I/E 1/5, tidal volume 6 ml/kg). CPR was organized into three periods associated to a specific tidal volume (period T0 to T5 => 5 minutes at 6 ml/kg - period T5 to T10 => 5 minutes at 12 ml/kg - period T10 to T15 => 5 minutes at 6 ml/kg). Blood gases were measured at each tidal volume change. Animals were sacrificed at the end of the protocol (i.e., low-flow period of 15 minutes) with a lethal dose of pentobarbital (60 mg.kg-1). |
| - *Sample size* | 1 animal for initial tests and 6 animals for the main study |
| - *Inclusion and exclusion criteria* | A first animal was used to test different levels of tidal volume. The six others were included in the main study and data analysis. |
| - *Randomization* | There was only one arm in the study with variation of tidal volumes during cardiopulmonary outcome. |
| - *Blinding* | The investigators were not blinded for the changes in tidal volumes during cardiopulmonary resuscitation. However, hemodynamic parameters were calculated automatically by a dedicated hemodynamic software (HEM version 4.2, Notocord, Croissy-sur-Seine, France). |
| - *Outcome measures* | Hemodynamic parameters were continuously recorded throughout the experimental protocol (HEM version 4.2, Notocord, Croissy-sur-Seine, France). Parameters continuously recorded were: airway pressure (mmHg), aortic blood pressure (mmHg), right atrial pressure (mmHg), intracranial pressure (mmHg), carotid blood flow (ml/min), CO2. Coronary perfusion pressure was calculated as aortic blood pressure minus right atrial pressure at end-decompression. Cerebral perfusion pressure was calculated as the mean value of arterial pressure minus intracranial pressure throughout cardiac compression. |
| - *Statistical methods* | Correlation was assessed between hemodynamic parameters and tidal volumes or distension ratio using a random effects linear model with each pig’s id as the random effect. All statistical tests were two-sided and results with p <0.05 were considered statistically significant. |
| - *Experimental animals* | Female pigs weighing 28±1 kg) |
| - *Experimental protocol* | We used 7 female pigs weighing 28±1 kg, including 1 animal for the initial test and 6 animals for the main study. They were hosted in Individual and contiguous boxes, allowing interaction between animals. A period of 7 to 10 days of acclimation was allowed before inclusion in the study. Then, swine were anesthetized with a mixture of tiletamine (10 mg.kg-1 i.v.), zolazepam (10 mg.kg-1, i.v.), propofol (10 mg.kg-1.h-1 i.v.) and methadone (0.3 mg/kg-1 i.m.). Animals were intubated and mechanically ventilated (Monnal T60, Air Liquide, Antony, France) in ACV mode (30% Oxygen, tidal volume 9 ml/kg, RR=20). Body temperature was controlled for a core temperature at 38°C. Animals were monitored by a five-lead electrocardiogram. Oxygen saturation (SpO2) and CO2 using the Monnal system (Irma CO2 probe Monnal, Masimo Corporation CA, USA) were recorded. Catheters were inserted into femoral vein and artery for the evaluation of aortic and right atrial pressure, respectively. An intracranial pressure probe was inserted after trepanation (Millar®, Houston, USA). A flow probe was implanted around the carotid artery for the continuous evaluation of the carotid blood flow (PS-Series Probes, Transonic, NY, USA). After a period of stabilization, animals were paralyzed by rocuronium (1 mg.kg-1). The mechanical compression device, a LUCASTM (Physio-control, Lund, Sweden) was placed in a controlled and secured position and was operated with default settings with a small active decompression (due to the suction cup. Cardiac arrest and cardiopulmonary resuscitation were then started as described above. |
| **RESULTS** | Data are reported in Tables (baseline data) and figures for all investigated parameters. |
